# Glucose Metabolism in Acute Kidney Injury and Kidney Repair

**DOI:** 10.3389/fmed.2021.744122

**Published:** 2021-11-29

**Authors:** Lu Wen, Ying Li, Siyao Li, Xiaoru Hu, Qingqing Wei, Zheng Dong

**Affiliations:** ^1^Hunan Key Laboratory of Kidney Disease and Blood Purification, Department of Nephrology, The Second Xiangya Hospital of Central South University, Changsha, China; ^2^Department of Cellular Biology and Anatomy, Medical College of Georgia at Augusta University, Augusta, GA, United States; ^3^Research Department, Charlie Norwood VA Medical Center, Augusta, GA, United States

**Keywords:** glucose metabolism, glycolysis (warburg effect), SGLTs, kidney injury and repair, renal fibrosis

## Abstract

The kidneys play an indispensable role in glucose homeostasis *via* glucose reabsorption, production, and utilization. Conversely, aberrant glucose metabolism is involved in the onset, progression, and prognosis of kidney diseases, including acute kidney injury (AKI). In this review, we describe the regulation of glucose homeostasis and related molecular factors in kidneys under normal physiological conditions. Furthermore, we summarize recent investigations about the relationship between glucose metabolism and different types of AKI. We also analyze the involvement of glucose metabolism in kidney repair after injury, including renal fibrosis. Further research on glucose metabolism in kidney injury and repair may lead to the identification of novel therapeutic targets for the prevention and treatment of kidney diseases.

## Introduction

In the 1930s, Bergman and Drury showed that the removal of the kidneys or blockade of their function markedly increased glucose requirement in rabbits, demonstrating the first evidence for the involvement of the kidneys in systemic glucose homeostasis. Today, the kidneys are known to contribute to glucose homeostasis *via* glucose reabsorption, production, and utilization. When the bloodstream flows through the kidneys, the bulk of glucose filtered by the glomeruli reenters circulation through reabsorption by renal tubules. In addition, when glycogen is exhausted after a lengthy fasting, the kidneys can produce glucose and release it into circulation through gluconeogenesis. Moreover, the kidneys are glucose consumers that utilize glucose as part of the energy source to support their reabsorptive activity and excrete metabolic wastes.

Acute insults such as hypoxia or ischemia, drugs or toxins, and infection can cause damage to the kidneys, resulting in acute kidney injury (AKI). The kidneys have the ability to repair, but if an injury is severe or recurring, the repair will be abnormal or maladaptive, resulting in renal fibrosis (1). There is accumulating evidence that glucose metabolism takes part in the progression of some kidney diseases, but its role in kidney injury and repair remains largely unknown. In this review, we discuss glucose homeostasis and its regulation in kidneys under normal physiological conditions, elucidate the role of glucose metabolism in the development and progression of AKI, and analyze its involvement in kidney repair, including renal fibrosis.

## Glucose Homeostasis in Normal Kidneys

**Figure 1:**
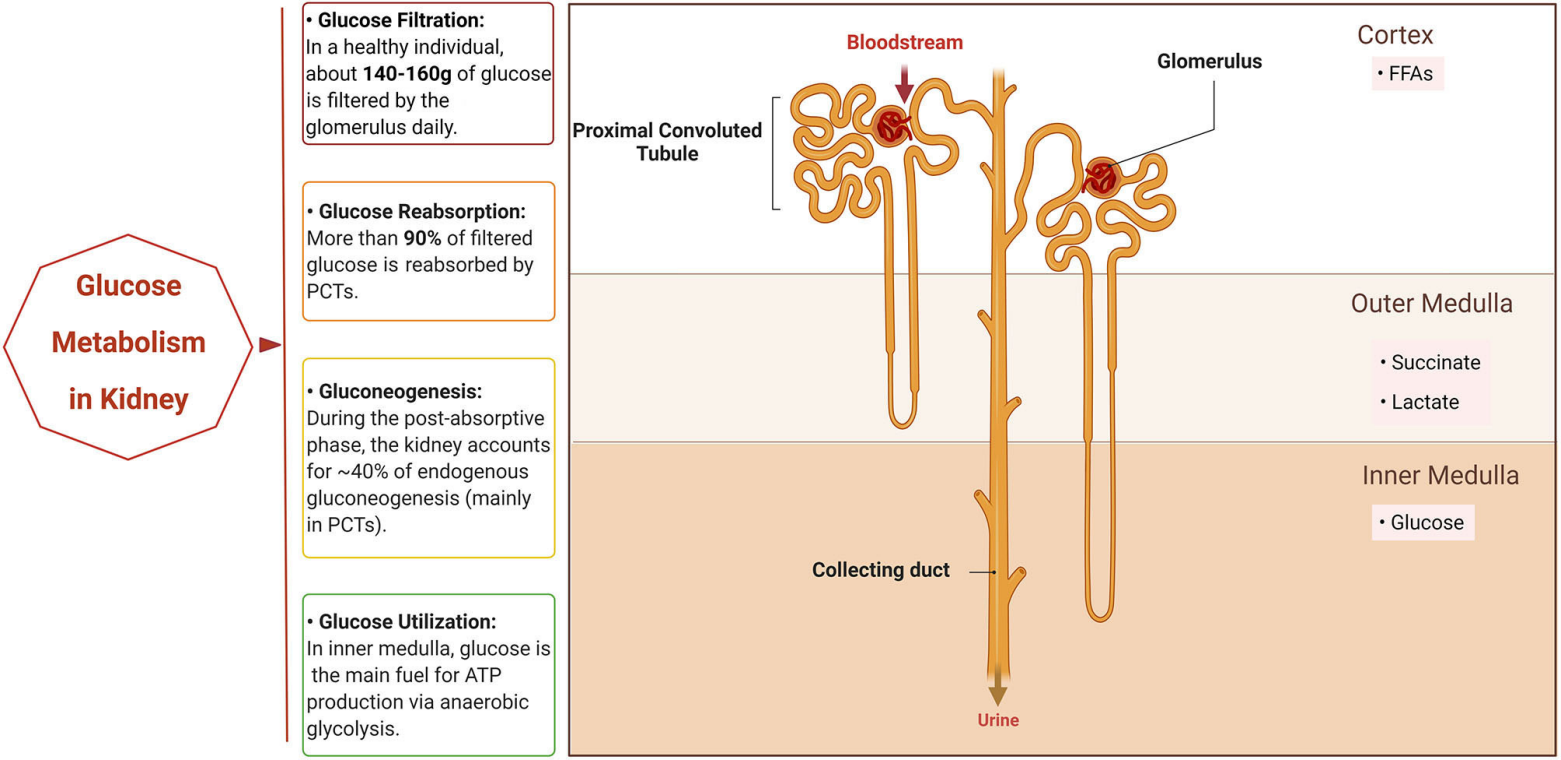
Glucose metabolism in normal kidney. The normal kidney is involved in systemic glucose metabolism mainly in three ways: reabsorption of glucose, production of glucose (gluconeogenesis), and utilization of glucose. When the bloodstream floods into the kidneys, all of the glucose in plasma passes through the glomerulus and most is then reabsorbed by the proximal convoluted tubules (PCTs). In addition, PCTs produce glucose through gluconeogenesis during the post-absorptive phase. Different segments of the nephron have their own preferable sources of fuels for energy based on oxygen availability. PCTs in the cortex prefer free fatty acids (FFAs) for respiration, while succinate and lactate are the main fuel for the outer medulla tubules and the inner medulla mainly utilizes glucose *via* anaerobic glycolysis to generate ATP. Created with BioRender.com.

Glucose is known to be the major carbon source for cellular biosynthesis and energy generation, which plays a significant role in cell growth (2). There are three ways that the kidneys take part in the homeostasis of systemic glucose. First, they reabsorb glucose into the bloodstream after glomerular filtration. Second, they synthesize and release glucose into circulation through gluconeogenesis. Finally, they make use of glucose from circulation to fuel cellular activities and functions (Figure 1). The traditional view elicited from net organ balance studies concluded that glucose homeostasis was mainly ensured by the liver, while the kidneys only played a significant role under acidotic conditions and after prolonged fasting (3). However, the kidneys are now considered to significantly contribute to systemic glucose homeostasis based on recent studies as elaborated below (4–6).

### Renal Glucose Reabsorption

In a healthy person with a normal estimated glomerular filtration rate (eGFR), about 140–160 g of glucose is filtered from the bloodstream by the glomerulus daily (7). Normally, the amount of glucose filtered by the kidneys rises linearly when the plasma glucose level increases because of its free filtration in the glomerulus (8). Most of the filtered glucose reenters the bloodstream through reabsorption by the proximal convoluted tubules (PCT) (7). At the cellular level, glucose is reabsorbed by the sodium–glucose co-transporters (SGLTs) that are located on the brush-border membrane of the PCTs. PCTs mainly depend on these transporters to transfer glucose across the plasma membrane since the plasma membrane of PTCs is impermeable to glucose (9). SGLT2 is a high-capacity, low-affinity SGLT located in the S1 and S2 segments of the PCT. By coupling with the transport of sodium in an adenosine triphosphate (ATP)-driven way, SGLT2 is responsible for the active transport of glucose against a concentration gradient, which accounts for the reabsorption of more than 90% of the glucose filtered by the glomerulus. In contrast, SGLT1 has a relatively lower capacity and higher affinity for glucose and is mainly expressed in the intestine and S3 segment of the PCTs, taking part in the absorption of glucose in the intestine and the reabsorption of the remaining part of glucose filtered by glomerulus. In addition to SGLTs, the facilitative glucose transporters, the GLUTs, which lie in the basolateral membrane of PCTs, also contribute to glucose transport. GLUT2, which is also known as SLC2A2, works synergistically with SGLT2 in the S1 segment of the renal tubule, while GLUT1, which is also known as SLC2A1, cooperates with SGLT1 in the S3 segment (9–12). When the plasma glucose concentration is < ~200 mg/dL, reabsorption of filtered glucose increases linearly since the reabsorptive capacity of the SGLTs is not yet saturated. Under such conditions, glucose is not present in the urine since the glucose filtered through the glomerulus is completely reabsorbed by the PCTs. The reabsorption curve is no longer linear when the glucose level exceeds the maximal amount that can be reabsorbed (the tubular maximum or T_max_) because the co-transporters are approaching saturation. This is referred to as the renal threshold and is usually seen at a plasma glucose concentration of ~200–250 mg/dL (9). When the plasma glucose concentration exceeds the renal threshold, for example, in diabetes, glucose leaks into the urine.

### Renal Gluconeogenesis

Two processes are involved in the endogenous production of glucose in circulation. One process is glycogenolysis, the process catalyzed by glucose-6-phosphatase that converts glycogen to glucose-6-phosphate (G6P) and subsequently hydrolyzes it into free glucose. The other process is gluconeogenesis, i.e., *de novo* synthesis of glucose from non-glucose precursors (13). Glycogenolysis mainly occurs in the liver, accounting for approximately 50% of circulating glucose in the post-absorptive state (14). Although glycogen synthesis and degradation have been noted in the medulla of kidney, it is widely believed that the kidneys are unable to release glucose through glycogenolysis because renal cells have negligible glucose-6-phosphatase activity (15). Gluconeogenesis consists of a series of enzyme-catalyzed reactions. Among them, there are four irreversible reactions that are catalyzed by four key enzymes: pyruvate carboxylase, phosphoenolpyruvate carboxykinase (PEPCK), fructose-1,6-bisphosphatase, and glucose-6-phosphatase. During the post-absorptive phase, the kidneys account for ~40% of endogenous gluconeogenesis, which primarily occurs in the PCTs and the proximal straight tubules since hub enzymes for gluconeogenesis are limited to the PCTs (16, 17). In humans, lactate, glutamine, alanine, and glycerol make up the main gluconeogenic substrates. However, it is worth noting that there are differences between the kidneys and the liver in their resources of gluconeogenic precursors. Lactate is the predominant gluconeogenic precursor in both organs, but glutamine is the preferential gluconeogenic substrate for the kidney, while alanine is preferentially used by the liver (18).

### Renal Glucose Utilization

The kidneys consume a significant portion of energy in our body to support filtration, reabsorption, and excretion. The preference for energy sources of kidney cells varies depending on their location along the nephron, specific ATP demands, and oxygen availability (19). For example, in the cortex of the kidneys, the PCT cells prefer free fatty acids (FFAs) rather than glucose as their fuel for respiration and tubular transport, while glucose is the preferred fuel substrate for the glomerulus and thin descending limbs. Because the cortex normally has sufficient oxygen supply, the cortical PCTs can use FFAs to produce more ATP although this may consume comparatively higher levels of oxygen than glucose oxidation (20). However, the oxygen supply decreases from the cortex to the medulla due to the shunting of cortical blood flow (21). In the outer medulla, succinate or lactate is the preferred source for respiration rather than glucose, while in the inner medulla, glucose is mainly converted to lactate *via* the process known as anaerobic glycolysis (16).

## Glucose Metabolism Regulation in Kidney

### Hormones

In healthy individuals, the plasma glucose concentration is maintained within a relatively narrow range of about 4.0–8.0 mM despite the wide fluctuations of food intake or exercise. This mainly depends on the regulation of hormones that can precisely adjust the endogenous production of glucose. Among those glucoregulatory factors, insulin, glucagon, and catecholamines are the most important acute glucoregulatory hormones and can alter the plasma glucose level in just a few minutes (22).

Insulin, a well-studied hormone, was discovered in 1921, but it was not until decades later that its mechanism of action was understood. Levine et al. later elucidated that the glucose-lowering effect of insulin was through augmenting membrane permeability to glucose rather than binding directly to glycolytic enzymes to modify their activity (23). In 1971, Freychet et al. proved the existence of a membrane receptor for insulin (24). A decade later, the insulin receptor (IR) was cloned contemporaneously by the Ebina and Ullrich groups (25, 26). Now it is clear that, by binding to the IRs, insulin can regulate and amplify intracellular insulin signaling, leading to the translocation of GLUT1 and GLUT4 to the plasma membrane for cellular glucose uptake and lowering of blood glucose. In addition, insulin signaling contributes to the regulation of different cellular processes through various pathways, such as the phospho-inositol-3 (PI3K) pathway, MAPK, AKT, and mTOR (27). For renal glucose metabolism, insulin is reported to attenuate glucose release through directly activating or deactivating enzymes in gluconeogenesis, as well as by reducing the availability of gluconeogenic substrates, and acts on gluconeogenic activators (14).

Glucagon is an important hormone in the regulation of liver glucose metabolism both in gluconeogenesis and in glycogenolysis, but its role in glucose metabolism in the kidney is negligible (28). Catecholamines can increase renal gluconeogenesis by directly stimulating cAMP-mediated key gluconeogenic enzymes and indirectly increasing the gluconeogenic precursors and stimulators (13). Other glucoregulatory hormones such as growth hormones, cortisol, and thyroid hormones also take part in the regulation of glucose homeostasis. The mechanism may be related to (1) influencing the sensitivity of the kidneys to the acute glucoregulatory hormones mentioned above; (2) modifying the activity of key enzymes that affect glycogen stores; or (3) affecting the availability of gluconeogenic precursors such as lactate, glycogen, and some amino acids (22).

### Substrates and Enzymes

As mentioned above, gluconeogenesis can synthesize glucose from various precursors, of which lactate is the most important. Due to a low oxygen supply, the distal tubules in the inner medulla mainly rely on anaerobic glycolysis to generate ATP, resulting in the generation of lactate. This, in turn, provides a substrate for cortical gluconeogenesis, leading to a corticomedullary glucose–lactate recycling loop (29).

Glycolysis, which converts glucose to pyruvate, is an important catabolic process in glucose metabolism. The rate of glycolytic flux is controlled precisely by key enzymes at different levels. During glycolysis, there are three vital enzymes, namely, hexokinase (HK), phosphofructokinase (PFK or PFK1), and pyruvate kinase (PK) that act synergistically. When glucose enters cells, it is immediately phosphorylated by HK, which is the first committed, rate-limiting step of glycolysis and drives all major pathways of glucose utilization. This rate-controlling step can maintain the concentration gradient of glucose, leading to continuous glucose uptake through the GLUTs. There are four isoforms of HK in mammalian cells, but the distribution, regulation, and function of the different isoforms in the kidney are unclear except for a description by Gall *et al* about endogenous HK II expression in the PCT (30–32). PFK or PFK-1 is another key enzyme during the glycolytic process that converts fructose-6-phosphate (F6P) to fructose-1,6-bisphosphate (F1,6P2) (33). While several intracellular metabolites modulate PFK, the most significant endogenous inhibitor and activator of PFK are ATP and fructose-2,6-bisphosphate (F2,6P2), respectively (34). Last, PK is a rate-limiting enzyme that catalyzes the irreversible process in converting phosphoenolpyruvate (PEP) to pyruvate in the last step of glycolysis. There are four isoforms of PK in mammals (L, R, M1, and M2), among which the M2 isoform (PKM2) expresses exclusively in rapidly proliferating tissues and is positively regulated by the upstream glycolytic intermediate F1,6P2 (35). There is emerging evidence that PKM2 is involved in the metabolic reprogramming progress of kidney diseases (36–38).

### Glucose Transporters

Sodium–glucose co-transporters are transmembrane proteins that belong to the SLC5 family of active glucose transporters, which contains 12 members (39). Among them, SGLT1 and SGLT2 are the major isoforms that have been studied comprehensively. As mentioned previously, SGLT1 accounts for most of the dietary glucose uptake in the intestine, while SGLT2 is related to the majority of glucose reabsorption in the kidneys. As a consequence, mutations in genes *SGLT1* and *SGLT2* can cause glucose/galactose malabsorption and glucosuria, separately (40). In humans, it is estimated that SGLT2 is responsible for the reabsorption of ~90% of tubular glucose, while the rest is handled by SGLT1 (41). Thus, the inhibition of SGLT2 can suppress renal glucose reabsorption to a great extent. Although glucosuria is connected with polyuria, polydipsia, nocturnal enuresis, and polyphagia, serious complications such as ascending urinary tract infections or impaired kidney function are rarely observed in individuals with *SGLT2* gene mutation, indicating that SGLT2 inhibitors could be developed as safe glucose-lowering drugs (40). As early as 1933, a natural substance called phloridzin from the root bark of apple trees was found to block the reabsorption of glucose in the kidneys (42). Subsequent studies demonstrated that phloridzin was a non-specific SGLT inhibitor that can cause diarrhea, dehydration, and other adverse reactions (40). Recently, several SGLT inhibitors have been discovered with high selectivity, high bioavailability, and safety. With the development of pharmaceutical technology, SGLT2 inhibitors are emerging and evolving. Specific SGLT2 inhibitors (canagliflozin, dapagliflozin, and empagliflozin) have entered clinical use in North America and Europe, while ipragliflozin, luseogliflozin, and tofogliflozin are being used in Japan. Other related SGLT inhibitors (e.g., ertugliflozin and sotagliflozin) are also under investigation (43).

## Regulation of Glucose Metabolism in Kidney Diseases

### The Warburg Effect

Initially described by Otto Warburg in 1924, the Warburg effect or aerobic glycolysis is defined as the induction of glycolysis in tissues or cells in the presence of oxygen, which plays a pivotal role in cancer metabolism (44). Compared to mitochondrial oxidative phosphorylation, aerobic glycolysis synthesizes fewer ATP molecules but produces ATP at a higher speed, i.e., generating more ATP in the same amount of time (45). In addition to energy production, aerobic glycolysis and the resulting metabolites are also involved in the regulation of various pathophysiology processes such as cell proliferation, extracellular matrix production, autophagy, and apoptosis (46). Although there is only a limited number of studies on the relationship of the Warburg effect and AKI (47), recent studies have provided compelling evidence that the Warburg effect contributes to the progression of kidney diseases such as polycystic kidney disease (PKD) and diabetic kidney disease (DKD) (38, 48, 49). Rowe et al. observed that aerobic glycolysis was a preferred source of energy rather than oxidative phosphorylation in *Pkd1*^−/−^ mouse embryonic fibroblasts. Consistently, aerobic glycolysis was enhanced in a murine model of PKD and human autosomal dominant polycystic kidney disease (ADPKD) kidneys, while the blockade of glycolysis with 2-deoxyglucose reduced the cystic index (48), suggesting a novel therapeutic paradigm for PKD. In diabetic mice and human patients, Sas et al. demonstrated the upregulation of glycolytic enzymes accompanied by increased glucose metabolism in kidneys (50). More recently, aerobic glycolysis was implicated in kidney injury caused by glucose fluctuation (51, 52). These studies suggest the involvement of aerobic glycolysis or the Warburg effect in the development of renal diseases.

### Glucose Metabolism in AKI

Aberrant glucose metabolism has been reported in the development of multiple human diseases, including cancers, type 2 diabetes mellitus (T2DM), and retinal disease (53–55). Similarly, the dysfunction of glucose metabolism may also contribute to the pathogenesis of AKI, and its role and regulation in AKI has attracted research interest in recent years (Table 1).

**Table 1:** Summary of the studies of glucose metabolism in acute kidney injury (AKI).

**AKI Categories**	**Involved substance in glucose metabolism**		**Models**	**Effects**	**Underlying mechanisms**	**References**
IRI-induced AKI	Glucose		Rat	Harmful	Active TLR-2, TLR4, and NF-kB and amplify upstream inflammatory response	(56)
	Hormones	Insulin	Rat/clinical trial	Protective	1. Phosphorylate insulin receptors, resulting in the improvement of endothelial function and increase in renal blood flow	(57, 58)
					2. Reduce iNOS activation	
					3. Active AKT, leading to the blockage of proapoptotic proteins such as BAD, BAX, and caspases	
	Substrates	FDP (F1,6P2)	Rat	Protective	1. Increase renal blood flow	(59–61)
					2. Maintain cellular ATP content	
					3. Inhibit ROS generation	
					4. Decrease LDH release	
		Pyruvate	Mouse	Protective	1. Increase ATP level	(62)
					2. Increase heme oxygenase 1 (HO-1) and IL-10	
					3. Decrease MCP-1	
		Lactate	Mouse/clinical trial	Harmful	Limit pyruvate synthesis because of the loss of lactate as a pyruvate precursor	(62–65)
	Enzymes	HK II	Rat/mouse	Protective	Reduce mitochondrial Bax accumulation and apoptosis	(30)
		PKM2	Mouse	Harmful	Increase oxidative stress	(66)
	Glucose	SGLT2	Rat/mouse	Harmful	1. Increase HIF1 expression	(67–70)
	transporters				2. Increase oxidative stress	
Cisplatin-induced AKI	Substrates	FDP (F1,6P2)	Rat	Protective	1. Act as a calcium chelator	(71)
					2. Attenuate the production of prostaglandin E and the expression of COX-2	
					3. Reduce the secretion of cytokines and the production of nitric oxide	
		Pyruvate	Rat	Protective	Act as a free radical scavenger	(72)
	Glucose transporters	SGLT2	Mouse/human kidney spheroids	Harmful	1. Decrease cisplatin uptake by renal tubular cells	(73–75)
					2. Activate AKT pathway	
					3. Impede glucose reabsorption	
Sepsis-induced AKI	Substrates	Lactate	Mouse/clinical retrospective study	Controversial	Active hydroxycarboxylic acid receptor 2(HCA2), lead to a decrease in proinflammatory cytokines	(76, 77)
	Enzymes	PKM2	Mouse	Harmful	Alter metabolic intermediates through the pentose phosphate pathway (PPP) to alleviate oxidative stress	(66)

#### Glucose Metabolism in Renal Ischemia–Reperfusion Injury

Renal ischemia/reperfusion injury (IRI) is a leading cause of AKI, which is often related to a variety of disease and treatment conditions such as renal vascular occlusion, kidney transplantation, and cardiac surgery (78). Complicated injurious factors are involved in renal IRI, including hypoxic injury, reactive oxygen species (ROS), mitochondrial dysfunction, and tubulointerstitial inflammation (79, 80). Dysregulation of glucose metabolism is closely related to renal IRI.

Hyperglycemia (HG) is involved in the amplification of the inflammatory response during renal IRI (56). Prolonged intravenous insulin-glucose administration could significantly accelerate the functional and histological recoveries of kidneys compared to the administration of glucose only during ischemic AKI in non-diabetic rats (57). Moreover, a randomized controlled clinical study indicated that a strict control of blood glucose levels with insulin reduced the morbidity, mortality, and requirement of dialysis or hemofiltration of AKI (58).

In addition to glucose, glycolysis metabolites also play roles in ischemic AKI. As early as the 1980s, Didlake et al. reported that fructose 1,6-diphosphate (FDP, also called fructose 1,6-bisphosphate, F1,6P2), a crucial intermediate in the glycolytic pathway, could prevent ischemic renal failure whether administered prior to the ischemic insult or during the post-ischemic reperfusion period in rats with bilateral renal artery occlusion (59, 60). More than a decade later, Antunes et al. demonstrated that FDP given before nephrectomy could attenuate renal cell injury in a cold ischemia rat model by maintaining cellular ATP content, decreasing lactate dehydrogenase (LDH) release, and preventing the microfilament disruption of PCT cells (61). Pyruvate is another key glycolytic metabolite sitting at the crossroad of anaerobic and aerobic energy production and can exert antioxidant and anti-inflammatory effects. Pyruvate depletion was detected in mice with unilateral ischemia and was accompanied by increased lactate and gluconeogenesis (pyruvate consumption) (62). Moreover, pyruvate therapy was also shown to mitigate functional damage in renal IRI. The underlying mechanisms may be related to increases in cytoprotective heme oxygenase 1 (HO-1) and IL-10, selective reduction of proinflammatory factors, and improved tissue ATP levels (62). Recently, Legouis et al. detected impaired glucose production and lactate clearance in patients with postoperative AKI and animals exposed to renal IRI. This altered glucose metabolism is a major determinant of systemic glucose and lactate levels and is strongly associated with mortality (63). Lan et al. further demonstrated that impaired mitochondrial function accompanied by enhanced glycolysis was a hallmark during renal IRI in rats, which was featured by higher levels of lactate and pyruvate and enhancement of HK activity (64). In addition, a multicenter cohort study showed that blood lactate was an independent predictive factor for AKI (65). As mentioned earlier, the kidneys mainly produce glucose from lactate through gluconeogenesis, particularly during fasting and stress conditions, making this organ a major systemic lactate dispersal site. When AKI occurs, the consumption of lactate decreases in kidneys, which may lead to lactate accumulation in blood.

Since accumulating evidence demonstrates that glycolysis is activated in ischemic AKI, the role of glycolytic enzymes in AKI has been questioned. Gall et al. found that total HK II content decreased and HK II was displaced from the mitochondria into the cytosol both *in vivo* (mouse) and *in vitro* in ischemic conditions, while the overexpression of HK II in opossum kidney PCT cells reduced mitochondrial Bax accumulation and apoptosis (30). On the contrary, Lan et al. detected increased protein levels of HK II, as well as other rate-limiting enzymes for glycolysis, including 6-phosphofructo-2-kinase/fructose-2,6-bisphosphatase 3 (PFKFB3) and PKM2 after renal IRI in rats (64). The discrepancy between these studies is likely related to the models and the times of analysis; Gall used mice and harvested the kidneys 3 hr after reperfusion, whereas Lan et al. tested rats at 3 days of reperfusion or later. In addition, using renal tubular epithelial cell-specific PKM2-knockout mice (*Pkm2*^−^/^−^) mice, Zhou et al. proved that *Pkm2*^−^/^−^ mice had better renal function and less histological tubular injury than wild-type (WT) mice following renal IRI or lipopolysaccharide (LPS)-induced endotoxic AKI (66). Several studies reported the protective effect of SGLT2 inhibitors in IRI-induced AKI (67–69), but a recent study showed that genetic deletion of SGLT2 from renal tubules did not protect against renal IRI (70). Therefore, the role of SGLTs in ischemic AKI remains to be established.

#### Glucose Metabolism in Cisplatin-Induced AKI

Cisplatin is a powerful antineoplastic agent that may induce nephrotoxicity. It causes tubular injury and cell death through multiple mechanisms, including DNA damage, oxidative stress, mitochondrial dysfunction, and inflammation (81, 82). Impairment of glucose metabolism has been implicated in cisplatin-induced AKI. Glucose was detected in the urine of mice at 48 hr after cisplatin administration. This metabolic alteration preceded the change of serum creatinine (83), and thus, it may be used as a biomarker of cisplatin-induced nephrotoxicity. F1,6P2 has shown a protective effect in the kidney during cisplatin nephrotoxicity (84). Azambuja et al. confirmed that rats that received cisplatin plus F1,6P2 presented a significantly lower level of serum creatinine and urea and less severe tubular necrosis compared to a cisplatin-only group, verifying the protective effect of F1,6P2 in cisplatin-induced AKI (71). Other glycolysis intermediates, such as pyruvate, have been reported to ameliorate cisplatin-induced AKI as well (72). Moreover, in 1992, Yanase et al. reported the decreased Na+-dependent D-glucose transport across renal brush-border membranes in cisplatin-induced AKI (85). This inhibition of Na+-coupled glucose uptake by cisplatin may result from direct chemical interactions with the SGLTs, leading to glucosuria in cisplatin-induced AKI (86). Intriguingly, a recent study found that canagliflozin, a SGLT2 inhibitor, could reduce histopathological injury in kidneys with cisplatin nephrotoxicity (73). We further demonstrated that the protective effect of canagliflozin in cisplatin-induced nephrotoxicity was related to decreased cisplatin uptake by renal tubular cells and the activation of the AKT pathway (74). In addition, Cohen et al. used organ-on-chip models, vascularized human kidney spheroids with integrated tissue-embedded microsensors for oxygen, glucose, lactate, and glutamine, to achieve a dynamic assessment of cellular metabolism and verified that empagliflozin (another SGLT2 inhibitor) could block cisplatin toxicity in the kidneys by impeding glucose reabsorption (75). Despite these studies, the molecular mechanisms underlying the changes of glucose metabolism in cisplatin-induced AKI remain largely unknown.

#### Glucose Metabolism in Sepsis-Associated AKI

Sepsis is characterized by organ dysfunction and failure resulting from the host's deleterious response to infection. It can lead to sepsis-associated acute kidney injury (SA-AKI), which contributes to high mortality and remains the most important cause of AKI (87, 88). In addition to renal hypotension and associated ischemia, inflammation and tubular injury are pathogenic factors in SA-AKI (87, 89). During SA-AKI, the lactate/pyruvate ratio rises in parallel with a significant decrease of renal cortex microvascular perfusion (90). It is worth noting that the role of lactate in SA-AKI remains controversial. Woolum et al. reported that thiamine could improve lactate clearance and reduce 28-day mortality (76). Conversely, Takakura et al. reported that lactate could negatively regulate macrophage activation and therefore acted as a negative feedback loop during sepsis to decrease the inflammatory response and improve the outcome (77). In addition to the glucose metabolites, sepsis can induce an early anabolic response in renal tissue that is characterized by a shift of metabolism toward aerobic glycolysis, followed by a decline in total renal ATP level (91, 92). Moreover, the deletion of PKM2 from mouse PCTs can protect against LPS-induced AKI. The mechanism is related to the SNO-CoA/SCoR system that shunts metabolic intermediates through the pentose phosphate pathway (PPP) to alleviate oxidative stress (66). Collectively, these findings suggest that glucose metabolism and its reprogramming are involved in SA-AKI but its precise role and regulation in injury development remains unknown.

#### Glucose Metabolism in Kidney Repair and Renal Fibrosis

After an acute insult in the kidneys, the surviving tubular cells undergo regeneration to restore the injured renal tubules (93). If the initial injury is severe, the repair is incomplete or maladaptive, eventually resulting in renal fibrosis and chronic kidney disease (CKD). In maladaptive repair after AKI, injured PCTs may become atrophic (94). Lan et al. found that these atrophic PCTs had elevated glycolysis after ischemic AKI, suggesting a role of glycolysis in maladaptive repair (64). Glycolytic enzymes were also detected to increase in CKD (64, 95, 96), although the role of glycolysis in CKD progression is less well defined. We demonstrated that the blockade of glycolysis with glycolysis inhibitors [dichloroacetate (DCA) or shikonin] could attenuate tubular apoptosis and the expression of extracellular matrix (fibronectin and collagen type I) in a mouse model of unilateral ureteral obstruction (UUO) (97). Similarly, Ding et al. demonstrated that the inhibition of aerobic glycolysis in UUO mice could suppress renal interstitial fibroblast activation and renal fibrosis (95). In contrast to these observations, the enhancement of glycolysis by TEPP-46, a PKM2 activator, decreased fibrotic protein expression in DKD in mice (38). In addition, using mice with inactivating mutations of the phosphorylation sites Ser468 and Ser485 in PFKFB2, a key glycolytic enzyme, Mardiana and colleagues showed that reduced glycolysis was associated with increased renal fibrosis in UUO and, to a less extent, in the model of folic acid nephropathy (98). These discrepant observations indicate that the role of glycolysis in renal fibrosis is more complex than originally thought and may depend on the cell types and its timing of activation. In addition to glycolysis, attention has been paid to the effects of HG on the PCT and how these effects promote renal fibrosis (99, 100). In diabetes, the glycemic threshold increases up to 200 mg/dL in patients, the mechanism of which is related to SGLTs in the tubular epithelium under conditions of chronic HG (101). Under HG, tubular cells undergo hypertrophy and apoptosis, which, to a great extent, contributes to renal fibrosis (102, 103). Thus, SGLTs can be an ideal target for renal fibrosis in such conditions. Indeed, recent studies have shown that SGLT2 inhibitors can attenuate fibrotic changes in diabetic mice (104, 105). Together, these studies indicate that the ability to regulate and maintain the appropriate level of glycolysis in the kidney is crucial for renal homeostasis, and anti-fibrosis strategies relying on the inhibition of glycolysis should depend on the type and location of the target cells.

## Discussion and Perspectives

Emerging evidence indicates that glucose and its metabolism play an inevitable role in AKI. The pharmacological intervention of glucose metabolism may reveal novel therapeutic strategies for AKI. However, there are still numerous questions to be answered. For example, what is the role of lactate in different types of AKI? What mechanisms lead to alterations in glucose metabolism in renal tubular cells in AKI? Reprogramming of glucose metabolism has been explored in a variety of cellular processes, such as tumor malignance, chronic inflammation, and cell proliferation, but the underlying mechanism remains obscure. Further understanding of the regulation and pathological effects of glucose metabolism in kidney injury and repair may lead to the discovery of new therapeutic approaches for AKI and prevention of the AKI-to-CKD transition.

## Author Contributions

LW and ZD contributed to the conceptualization, design, and outline of this review. LW prepared the original draft with figures. LW, YL, SL, XH, QW, and ZD contributed to the revision and editing. All authors have read and agreed to the published version of the manuscript.

## Funding

The authors were supported by grants from the National Institutes of Health of USA (DK058831 and DK087843) and Department of Veterans Administration of USA (BX000319).

## Conflict of Interest

The authors declare that the research was conducted in the absence of any commercial or financial relationships that could be construed as a potential conflict of interest.

## Publisher's Note

All claims expressed in this article are solely those of the authors and do not necessarily represent those of their affiliated organizations, or those of the publisher, the editors and the reviewers. Any product that may be evaluated in this article, or claim that may be made by its manufacturer, is not guaranteed or endorsed by the publisher.

## References

[1] He L Wei Q Liu J Yi M Liu Y Liu H. AKI on CKD: heightened injury, suppressed repair, and the underlying mechanisms. Kidney Int. (2017) 92:1071–83. 10.1016/j.kint.2017.06.030 28890325 PMC5683166

[2] Mulukutla BC Yongky A Le T Mashek DG Hu. Regulation of glucose metabolism - a perspective from cell bioprocessing. Trends Biotechnol. (2016) 34:638–51. 10.1016/j.tibtech.2016.04.012 27265890

[3] Cano N. Bench-to-bedside review: Glucose production from the kidney. Crit Care. (2002) 6:317–21. 10.1186/cc1517 12225606 PMC137457

[4] Hughes CB Mussman GM Ray P Bunn RC Cornea V Thrailkill KM. Impact of an SGLT2-loss of function mutation on renal architecture, histology, and glucose homeostasis. Cell Tissue Res. (2021) 384:527–43. 10.1007/s00441-020-03358-8 33409652

[5] Legouis D Faivre A Cippa PE de Seigneux S. Renal gluconeogenesis: an underestimated role of the kidney in systemic glucose metabolism. Nephrol Dial Transplant. 2020:302. 10.1093/ndt/gfaa302 33247734

[6] Kaneko K Soty M Zitoun C Duchampt A Silva M Philippe E. The role of kidney in the inter-organ coordination of endogenous glucose production during fasting. Mol Metab. (2018) 16:203–12. 10.1016/j.molmet.2018.06.010 29960865 PMC6157617

[7] Vallon V Thomson SC. Targeting renal glucose reabsorption to treat hyperglycaemia: the pleiotropic effects of SGLT2 inhibition. Diabetologia. (2017) 60:215–25. 10.1007/s00125-016-4157-3 27878313 PMC5884445

[8] Alsahli M Gerich JE. Renal glucose metabolism in normal physiological conditions and in diabetes. Diabetes Res Clin Pract. (2017) 133:1–9. 10.1016/j.diabres.2017.07.033 28866383

[9] Chao EC Henry RR. SGLT2 inhibition–a novel strategy for diabetes treatment. Nat Rev Drug Discov. (2010) 9:551–9. 10.1038/nrd3180 20508640

[10] Liu JJ Lee T DeFronzo RA. Why Do SGLT2 inhibitors inhibit only 30-50% of renal glucose reabsorption in humans? Diabetes. (2012) 61:2199–204. 10.2337/db12-0052 22923645 PMC3425428

[11] Sabolić I Skarica M Gorboulev V Ljubojević M Balen D Herak-Kramberger CM. Rat renal glucose transporter SGLT1 exhibits zonal distribution and androgen-dependent gender differences. Am J Physiol Renal Physiol. (2006) 290:F913–26. 10.1152/ajprenal.00270.2005 16204409

[12] Bakris GL Fonseca VA Sharma K Wright EM. Renal sodium-glucose transport: role in diabetes mellitus and potential clinical implications. Kidney Int. (2009) 75:1272–77. 10.1038/ki.2009.87 19357717

[13] Stumvoll M Meyer C Mitrakou A Nadkarni V Gerich JE. Renal glucose production and utilization: New aspects in humans. Diabetologia. (1997) 40:749–57. 10.1007/s001250050745 9243094

[14] Mitrakou A. Kidney: its impact on glucose homeostasis and hormonal regulation. Diabetes Research and Clinical Practice. (2011) 93:S66–S72. 10.1016/S0168-8227(11)70016-X 21864754

[15] Guder WG Ross BD. Enzyme distribution along the nephron. Kidney Int. (1984) 26:101–11. 10.1038/ki.1984.143 6094907

[16] Ross BD Espinal J Silva P. Glucose metabolism in renal tubular function. Kidney Int. (1986) 29:54–67. 10.1038/ki.1986.8 3515015

[17] Schoolwerth AC Smith BC Culpepper RM. Renal gluconeogenesis. Mineral and electrolyte metabolism. (1988) 14:347–61. 3068502

[18] Stumvoll M Meyer C Perriello G Kreider M Welle S Gerich J. Human kidney and liver gluconeogenesis: evidence for organ substrate selectivity. Am J Physiol. (1998) 274:E817–26. 10.1152/ajpendo.1998.274.5.E817 9612239

[19] Sullivan MA Forbes JM. Glucose and glycogen in the diabetic kidney: heroes or villains? EBioMedicine. (2019) 47:590–7. 10.1016/j.ebiom.2019.07.067 31405756 PMC6796499

[20] Forbes JM Thorburn DR. Mitochondrial dysfunction in diabetic kidney disease. Nat Rev Nephrol. (2018) 14:291–312. 10.1038/nrneph.2018.9 29456246

[21] Chen Y Fry BC Layton AT. modeling glucose metabolism in the kidney. Bull Math Biol. (2016) 78:1318–36. 10.1007/s11538-016-0188-7 27371260 PMC5431085

[22] Gerich JE. Physiology of glucose homeostasis. Diabetes Obes Metab. (2000) 2:345–50. 10.1046/j.1463-1326.2000.00085.x 11225963

[23] Levine R Goldstein MS Huddlestun B Klein SP. Action of insulin on the 'permeability' of cells to free hexoses, as studied by its effect on the distribution of galactose. Am J Physiol. (1950) 163:70–6. 10.1152/ajplegacy.1950.163.1.70 14771275

[24] Freychet P Roth J Neville DM. Insulin receptors in the liver: specific binding of (125 I)insulin to the plasma membrane and its relation to insulin bioactivity. Proc Natl Acad Sci U S A. (1971) 68:1833–7. 10.1073/pnas.68.8.1833 4331561 PMC389303

[25] Ebina Y Ellis L Jarnagin K Edery M Graf L Clauser E. The human insulin receptor cDNA: the structural basis for hormone-activated transmembrane signalling. Cell. (1985) 40:747–58. 10.1016/0092-8674(85)90334-4 2859121

[26] Ullrich A Bell JR Chen EY Herrera R Petruzzelli LM Dull TJ. Human insulin receptor and its relationship to the tyrosine kinase family of oncogenes. Nature. (1985) 313 (6005):756–61. 10.1038/313756a0 2983222

[27] Kuczkowski A Brinkkoetter PT. Metabolism and homeostasis in the kidney: metabolic regulation through insulin signaling in the kidney. Cell Tissue Res. (2017) 369:199–210. 10.1007/s00441-017-2619-7 28413863

[28] Stumvoll M Meyer C Kreider M Perriello G Gerich J. Effects of glucagon on renal and hepatic glutamine gluconeogenesis in normal postabsorptive humans. Metabolism. (1998) 47:1227–32. 10.1016/S0026-0495(98)90328-6 9781626

[29] Bartlett S Espinal J Janssens P Ross BD. The influence of renal function on lactate and glucose metabolism. Biochem J. (1984) 219:73–8. 10.1042/bj2190073 6721865 PMC1153449

[30] Gall JM Wong V Pimental DR Havasi A Wang Z Pastorino JG. Hexokinase regulates Bax-mediated mitochondrial membrane injury following ischemic stress. Kidney Int. (2011) 79:1207–16. 10.1038/ki.2010.532 21430642 PMC3361076

[31] Nederlof R Eerbeek O Hollmann MW Southworth R Zuurbier CJ. Targeting hexokinase II to mitochondria to modulate energy metabolism and reduce ischaemia-reperfusion injury in heart. Br J Pharmacol. (2014) 171:2067–79. 10.1111/bph.12363 24032601 PMC3976622

[32] Robey RB. Hexokinase: a novel sugar kinase coupled to renal epithelial cell survival. Kidney Int. (2011) 79:1163–5. 10.1038/ki.2011.20 21566638

[33] Shi L Pan H Liu Z Xie J Han W. Roles of PFKFB3 in cancer. Signal Transduct Target Ther. (2017) 2:17044. 10.1038/sigtrans.2017.44 29263928 PMC5701083

[34] Al Hasawi N Alkandari MF Luqmani YA. Phosphofructokinase: a mediator of glycolytic flux in cancer progression. Crit Rev Oncol Hematol. (2014) 92:312–21. 10.1016/j.critrevonc.2014.05.007 24910089

[35] Mulukutla BC Khan S Lange A Hu WS. Glucose metabolism in mammalian cell culture: new insights for tweaking vintage pathways. Trends Biotechnol. (2010) 28:476–84. 10.1016/j.tibtech.2010.06.005 20691487

[36] Yuan Q Miao J Yang Q Fang L Fang Y Ding H. Role of pyruvate kinase M2-mediated metabolic reprogramming during podocyte differentiation. Cell Death Dis. (2020) 11:355. 10.1038/s41419-020-2481-5 32393782 PMC7214446

[37] Liu H Takagaki Y Kumagai A Kanasaki K Koya D. The PKM2 activator TEPP-46 suppresses kidney fibrosis via inhibition of the EMT program and aberrant glycolysis associated with suppression of HIF-1α accumulation. J Diabet Investigat. (2020) 12:697–709. 10.1111/jdi.13478 33314682 PMC8089020

[38] Qi W Keenan HA Li Q Ishikado A Kannt A Sadowski T. Pyruvate kinase M2 activation may protect against the progression of diabetic glomerular pathology and mitochondrial dysfunction. Nat Med. (2017) 23:753–62. 10.1038/nm.4328 28436957 PMC5575773

[39] Wright EM Loo DD Hirayama BA Turk E. Surprising versatility of Na+-glucose cotransporters: SLC5. Physiology. (2004) 19:370–6. 10.1152/physiol.00026.2004 15546855

[40] Rieg T Vallon V. Development of SGLT1 and SGLT2 inhibitors. Diabetologia. (2018) 61:2079–86. 10.1007/s00125-018-4654-7 30132033 PMC6124499

[41] Gallo LA Wright EM Vallon V. Probing SGLT2 as a therapeutic target for diabetes: basic physiology and consequences. Diab Vasc Dis Res. (2015) 12:78–89. 10.1177/1479164114561992 25616707 PMC5886707

[42] Chasis H Jolliffe N Smith HW. The action of phlorizin on the excretion of glucose, xylose, sucrose, creatinine and urea by man. J Clin Invest. (1933) 12:1083–90. 10.1172/JCI100559 16694183 PMC435965

[43] Heerspink HJL Kosiborod M Inzucchi SE Cherney DZI. Renoprotective effects of sodium-glucose cotransporter-2 inhibitors. Kidney Int. (2018) 94:26–39. 10.1016/j.kint.2017.12.027 29735306

[44] Zhang G Darshi M Sharma K. The Warburg Effect in Diabetic Kidney Disease. Semin Nephrol. (2018) 38:111–20. 10.1016/j.semnephrol.2018.01.002 29602394 PMC5973839

[45] Chen Z Liu M Li L Chen L. Involvement of the Warburg effect in non-tumor diseases processes. J Cell Physiol. (2018) 233:2839–49. 10.1002/jcp.25998 28488732

[46] Lunt SY Vander Heiden MG. Aerobic glycolysis: meeting the metabolic requirements of cell proliferation. Annu Rev Cell Dev Biol. (2011) 27:441–64. 10.1146/annurev-cellbio-092910-154237 21985671

[47] Tan C Gu J Li T Chen H Liu K Liu M. Inhibition of aerobic glycolysis alleviates sepsis-induced acute kidney injury by promoting lactate/Sirtuin 3/AMPKregulated autophagy. Int J Mol Med. (2021) 47:4852. 10.3892/ijmm.2021.4852 33448325 PMC7849980

[48] Rowe I Chiaravalli M Mannella V Ulisse V Quilici G Pema M. Defective glucose metabolism in polycystic kidney disease identifies a new therapeutic strategy. Nat Med. (2013) 19:488–93. 10.1038/nm.3092 23524344 PMC4944011

[49] Beck Gooz M Maldonado EN Dang Y Amria MY Higashiyama S Abboud HE. ADAM17 promotes proliferation of collecting duct kidney epithelial cells through ERK activation and increased glycolysis in polycystic kidney disease. Am J Physiol Renal Physiol. (2014) 307:F551–9. 10.1152/ajprenal.00218.2014 24899059 PMC4154111

[50] Sas KM Kayampilly P Byun J Nair V Hinder LM Hur J. Tissue-specific metabolic reprogramming drives nutrient flux in diabetic complications. JCI Insight. (2016) 1:e86976. 10.1172/jci.insight.86976 27699244 PMC5033761

[51] Xu WL Liu S Li N Ye LF Zha M Li CY. Quercetin antagonizes glucose fluctuation induced renal injury by inhibiting aerobic glycolysis via hif-1alpha/mir-210/iscu/fes pathway. Front Med. (2021) 8:656086. 10.3389/fmed.2021.656086 33748166 PMC7969708

[52] Fu X Zhang J Huang X Mo Z Sang Z Duan W. Curcumin antagonizes glucose fluctuation-induced renal injury by inhibiting aerobic glycolysis via the mir-489/ldha pathway. Mediators Inflamm. (2021) 21:6104529. 10.1155/2021/6104529 34456629 PMC8387199

[53] Agius L Ford BE Chachra SS. The metformin mechanism on gluconeogenesis and ampk activation: the metabolite perspective. Int J Mol Sci. (2020) 21:240. 10.3390/ijms21093240 32375255 PMC7247334

[54] Jiang P Du W Wu M. Regulation of the pentose phosphate pathway in cancer. Protein Cell. (2014) 5:592–602. 10.1007/s13238-014-0082-8 25015087 PMC4112277

[55] Joyal JS Sun Y Gantner ML Shao Z Evans LP Saba N. Retinal lipid and glucose metabolism dictates angiogenesis through the lipid sensor Ffar1. Nat Med. (2016) 22:439–45. 10.1038/nm.4059 26974308 PMC4823176

[56] Jun JH Song JW Shin EJ Kwak YL Choi N Shim JK. Ethyl pyruvate is renoprotective against ischemia-reperfusion injury under hyperglycemia. J Thorac Cardiovasc Surg. (2018) 155:1650–1658. 10.1016/j.jtcvs.2017.10.069 29195627

[57] Melo RS Visona I Almeida WS Campos AH. Glucose-insulin infusion reduces kidney injury in an experimental model of ischemic nephropathy. Am J Nephrol. (2010) 32:603–9. 10.1159/000319622 21099217

[58] Schetz M Vanhorebeek I Wouters PJ Wilmer A Van den Berghe G. Tight blood glucose control is renoprotective in critically ill patients. J Am Soc Nephrol. (2008) 19:571–8. 10.1681/ASN.2006101091 18235100 PMC2391044

[59] Didlake R Kirchner KA Lewin J Bower JD Markov A. Protection from ischemic renal injury by fructose-1,6-diphosphate infusion in the rat. Circulat Shock. (1985) 16:205–12. 4053295

[60] Didlake R Kirchner KA Lewin J Bower JD Markov AK. Attenuation of ischemic renal injury with fructose 1,6-diphosphate. J Surg Res. (1989) 47:220–6. 10.1016/0022-4804(89)90111-X 2770278

[61] Antunes N Martinusso CA Takiya CM da Silva AJ de Ornellas JF Elias PR. Fructose-1,6 diphosphate as a protective agent for experimental ischemic acute renal failure. Kidney Int. (2006) 69:68–72. 10.1038/sj.ki.5000013 16374425

[62] Zager RA Johnson AC Becker K. Renal cortical pyruvate depletion during AKI. J Am Soc Nephrol. (2014) 25:998–1012. 10.1681/ASN.2013070791 24385590 PMC4005311

[63] Legouis D Ricksten SE Faivre A Verissimo T Gariani K Verney C. Altered proximal tubular cell glucose metabolism during acute kidney injury is associated with mortality. Nat Metab. (2020) 2:732–43. 10.1038/s42255-020-0238-1 32694833

[64] Lan R Geng H Singha PK Saikumar P Bottinger EP Weinberg JM. Mitochondrial pathology and glycolytic shift during proximal tubule atrophy after ischemic AKI. J Am Soc Nephrol. (2016) 27:3356–67. 10.1681/ASN.2015020177 27000065 PMC5084876

[65] Harrois A Soyer B Gauss T Hamada S Raux M Duranteau J. Prevalence and risk factors for acute kidney injury among trauma patients: a multicenter cohort study. Crit Care. (2018) 22:344. 10.1186/s13054-018-2265-9 30563549 PMC6299611

[66] Zhou HL Zhang R Anand P Stomberski CT Qian Z Hausladen A. Metabolic reprogramming by the S-nitroso-CoA reductase system protects against kidney injury. Nature. (2019) 565:96–100. 10.1038/s41586-018-0749-z 30487609 PMC6318002

[67] Chang YK Choi H Jeong JY Na KR Lee KW Lim BJ. Dapagliflozin, SGLT2 Inhibitor, Attenuates Renal Ischemia-Reperfusion Injury. PLoS One. (2016) 11:e0158810. 10.1371/journal.pone.0158810 27391020 PMC4938401

[68] Rezq S Nasr AM Shaheen A Elshazly SM. Doxazosin down-regulates sodium-glucose cotransporter-2 and exerts a renoprotective effect in rat models of acute renal injury. Basic Clin Pharmacol Toxicol. (2020) 126:413–423. 10.1111/bcpt.13371 31788938

[69] Nasr AM Rezq S Shaheen A Elshazly SM. Renal protective effect of nebivolol in rat models of acute renal injury: role of sodium glucose co-transporter 2. Pharmacol Rep. (2020) 72:956–68. 10.1007/s43440-020-00059-5 32128711

[70] Nespoux J Patel R Zhang H Huang W Freeman B Sanders PW. Gene knockout of the Na(+)-glucose cotransporter SGLT2 in a murine model of acute kidney injury induced by ischemia-reperfusion. Am J Physiol Renal Physiol. (2020) 318:F1100–12. 10.1152/ajprenal.00607.2019 32116018 PMC7294332

[71] Azambuja AA Lunardelli A Nunes FB Gaspareto PB Donadio MV Poli de Figueiredo CE. Effect of fructose-1,6-bisphosphate on the nephrotoxicity induced by cisplatin in rats. Inflammation. (2011) 34:67–71. 10.1007/s10753-010-9212-5 20419391

[72] Kelle I Akkoc H Tunik S Nergiz Y Erdinc M Erdinc L. Protective effects of ethyl pyruvate in cisplatin-induced nephrotoxicity. Biotechnol Biotechnol Equip. (2014) 28:674–80. 10.1080/13102818.2014.942489 26019553 PMC4433950

[73] Abdelrahman AM Al Suleimani Y Shalaby A Ashique M Manoj P Nemmar A. Effect of canagliflozin, a sodium glucose co-transporter 2 inhibitor, on cisplatin-induced nephrotoxicity in mice. Naunyn Schmiedebergs Arch Pharmacol. (2019) 392:45–53. 10.1007/s00210-018-1564-7 30206656

[74] Song Z Zhu J Wei Q Dong G Dong Z. Canagliflozin reduces cisplatin uptake and activates Akt to protect against cisplatin-induced nephrotoxicity. Am J Physiol Renal Physiol. (2020) 318:F1041–52. 10.1152/ajprenal.00512.2019 32150448 PMC7191450

[75] Cohen A Ioannidis K Ehrlich A Regenbaum S Cohen M Ayyash M. Mechanism and reversal of drug-induced nephrotoxicity on a chip. Sci Transl Med. (2021) 13:582. 10.1126/scitranslmed.abd6299 33627489 PMC8897043

[76] Woolum JA Abner EL Kelly A Thompson Bastin ML Morris PE Flannery AH. Effect of thiamine administration on lactate clearance and mortality in patients with septic shock. Crit Care Med. (2018) 46:1747–52. 10.1097/CCM.0000000000003311 30028362

[77] Takakura A Zandi-Nejad K. Lactate-induced activation of HCA2 improves survival in mice with sepsis. FASEB J. (2019) 33:7625–34. 10.1096/fj.201801982R 30951370

[78] Wei Q Dong Z. Mouse model of ischemic acute kidney injury: technical notes and tricks. Am J Physiol Renal Physiol. (2012) 303:F1487–94. 10.1152/ajprenal.00352.2012 22993069 PMC3532486

[79] Bonventre JV Yang L. Cellular pathophysiology of ischemic acute kidney injury. J Clin Invest. (2011) 121:4210–21. 10.1172/JCI45161 22045571 PMC3204829

[80] Tang C Han H Liu Z Liu Y Yin L Cai J. Activation of BNIP3-mediated mitophagy protects against renal ischemia-reperfusion injury. Cell Death Dis. (2019) 10:677. 10.1038/s41419-019-1899-0 31515472 PMC6742651

[81] Freitas-Lima LC Budu A Arruda AC Perilhao MS Barrera-Chimal J Araujo RC. PPAR-alpha deletion attenuates cisplatin nephrotoxicity by modulating renal organic transporters MATE-1 and OCT-2. Int J Mol Sci. (2020) 21. 10.3390/ijms21197416 33049997 PMC7582648

[82] Li S Wen L Hu X Wei Q Dong Z. HIF in nephrotoxicity during cisplatin chemotherapy: regulation, function and therapeutic potential. Cancers (Basel). (2021) 13:180. 10.3390/cancers13020180 33430279 PMC7825709

[83] Portilla D Li S Nagothu KK Megyesi J Kaissling B Schnackenberg L. Metabolomic study of cisplatin-induced nephrotoxicity. Kidney Int. (2006) 69:2194–204. 10.1038/sj.ki.5000433 16672910

[84] Alva N Alva R Carbonell T. Fructose 1,6-Bisphosphate: A summary of its cytoprotective mechanism. Curr Med Chem. (2016) 23:4396–4417. 10.2174/0929867323666161014144250 27758716

[85] Yanase M Uyama O Nakanishi T Shiratsuki N Sugita M. Decreased sodium dependent D-glucose transport across renal brush-border membranes in cis-diamminedichloride platinum induced acute renal failure. Ren Fail. (1992) 14:23–30. 10.3109/08860229209039113 1561386

[86] Potdevin S Courjault-Gautier F Ripoche P Toutain HJ. Similar effects of cis-diamminedichloroplatinum(II) and cis-diammine-1,1-cyclobutanedicarboxylatoplatinum(II) on sodium-coupled glucose uptake in renal brush-border membrane vesicles. Arch Toxicol. (1998) 72:663–70. 10.1007/s002040050558 9851683

[87] Poston JT Koyner JL. Sepsis associated acute kidney injury. BMJ. (2019) 364:k4891. 10.1136/bmj.k4891 30626586 PMC6890472

[88] Prowle JR. Sepsis-Associated AKI. Clin J Am Soc Nephrol. (2018) 13:339–342. 10.2215/CJN.07310717 29070523 PMC5967431

[89] Langenberg C Gobe G Hood S May CN Bellomo R. Renal histopathology during experimental septic acute kidney injury and recovery. Crit Care Med. (2014) 42:e58–67. 10.1097/CCM.0b013e3182a639da 24126439

[90] Chvojka J Sykora R Krouzecky A Radej J Varnerova V Karvunidis T. Renal haemodynamic, microcirculatory, metabolic and histopathological responses to peritonitis-induced septic shock in pigs. Crit Care. (2008) 12:R164. 10.1186/cc7164 19108740 PMC2646329

[91] Waltz P Carchman E Gomez H Zuckerbraun B. Sepsis results in an altered renal metabolic and osmolyte profile. J Surg Res. (2016) 202:8–12. 10.1016/j.jss.2015.12.011 27083942

[92] Smith JA Stallons LJ Schnellmann RG. Renal cortical hexokinase and pentose phosphate pathway activation through the EGFR/Akt signaling pathway in endotoxin-induced acute kidney injury. Am J Physiol Renal Physiol. (2014) 307:F435–44. 10.1152/ajprenal.00271.2014 24990892 PMC4137133

[93] Ferenbach DA Bonventre JV. Mechanisms of maladaptive repair after AKI leading to accelerated kidney ageing and CKD. Nat Rev Nephrol. (2015) 11:264–76. 10.1038/nrneph.2015.3 25643664 PMC4412815

[94] Yang L Besschetnova TY Brooks CR Shah JV Bonventre JV. Epithelial cell cycle arrest in G2/M mediates kidney fibrosis after injury. Nat Med. (2010) 16:535–43. 10.1038/nm.2144 20436483 PMC3928013

[95] Ding H Jiang L Xu J Bai F Zhou Y Yuan Q. Inhibiting aerobic glycolysis suppresses renal interstitial fibroblast activation and renal fibrosis. Am J Physiol Renal Physiol. (2017) 313:F561–F575. 10.1152/ajprenal.00036.2017 28228400

[96] Kang HM Ahn SH Choi P Ko YA Han SH Chinga F. Defective fatty acid oxidation in renal tubular epithelial cells has a key role in kidney fibrosis development. Nat Med. (2015) 21:37–46. 10.1038/nm.3762 25419705 PMC4444078

[97] Wei Q Su J Dong G Zhang M Huo Y Dong Z. Glycolysis inhibitors suppress renal interstitial fibrosis via divergent effects on fibroblasts and tubular cells. Am J Physiol Renal Physiol. (2019) 316:F1162–72. 10.1152/ajprenal.00422.2018 30969803 PMC6620587

[98] Lee M Harley G Katerelos M Gleich K Sullivan MA Laskowski A. Mutation of regulatory phosphorylation sites in PFKFB2 worsens renal fibrosis. Sci Rep. (2020) 10:14531. 10.1038/s41598-020-71475-z 32884050 PMC7471692

[99] Sireesha M Sambasivan V Kumar VK Radha S Raj AY Qurratulain H. Relevance of insulin-like growth factor 2 in the etiopathophysiology of diabetic nephropathy: possible roles of phosphatase and tensin homolog on chromosome 10 and secreted protein acidic and rich in cysteine as regulators of repair. J Diabetes. (2009) 1:118–24. 10.1111/j.1753-0407.2009.00025.x 20929508

[100] Dou L Jourde-Chiche N. Endothelial toxicity of high glucose and its by-products in diabetic kidney disease. Toxins. (2019) 11:10. 10.3390/toxins11100578 31590361 PMC6833015

[101] Alicic RZ Johnson EJ Tuttle KR. SGLT2 inhibition for the prevention and treatment of diabetic kidney disease: a review. Am J Kidney Dis. (2018) 72:267–77. 10.1053/j.ajkd.2018.03.022 29866460

[102] Brezniceanu ML Liu F Wei CC Chenier I Godin N Zhang SL. Attenuation of interstitial fibrosis and tubular apoptosis in db/db transgenic mice overexpressing catalase in renal proximal tubular cells. Diabetes. (2008) 57:451–9. 10.2337/db07-0013 17977949

[103] Ma Z Li L Livingston MJ Zhang D Mi Q Zhang M. p53/microRNA-214/ULK1 axis impairs renal tubular autophagy in diabetic kidney disease. J Clin Invest. (2020) 130:5011–5026. 10.1172/JCI135536 32804155 PMC7456229

[104] Zhang Y Nakano D Guan Y Hitomi H Uemura A Masaki T. A sodium-glucose cotransporter 2 inhibitor attenuates renal capillary injury and fibrosis by a vascular endothelial growth factor-dependent pathway after renal injury in mice. Kidney Int. (2018) 94:524–535. 10.1016/j.kint.2018.05.002 30045814

[105] Cai T Ke Q Fang Y Wen P Chen H Yuan Q. Sodium-glucose cotransporter 2 inhibition suppresses HIF-1alpha-mediated metabolic switch from lipid oxidation to glycolysis in kidney tubule cells of diabetic mice. Cell Death Dis. (2020) 11:390. 10.1038/s41419-020-2544-7 32444604 PMC7242894

